# Supplementary material for: A Critical View of the Application of the APEX Software (Aqueous Photochemistry of Environmentally-Occurring Xenobiotics) to Predict Photoreaction Kinetics in Surface Freshwaters
Source: Molecules. 2019 Dec 18;25(1):9. doi: 10.3390/molecules25010009 (PMC7017383; doi:10.3390/molecules25010009)
Supplement: Supplementary file 1 [file molecules-25-00009-s001.zip › 3.2.4_gcc-4.4.0.zip!/3.2.4_gcc-4.4.0/3.2.4_gcc-4.4.0/doc/octave/html/interpreter/Ordinary-Differential-Equations.html]

Ordinary Differential Equations - Untitled


Next: Differential-Algebraic Equations,
Up: Differential Equations


---

### 23.1 Ordinary Differential Equations

The function `lsode` can be used to solve ODEs of the form

```
     dx
     -- = f (x, t)
     dt
```

using Hindmarsh's ODE solver Lsode.

— Loadable Function: [x, istate, msg] = **lsode** (fcn, x\_0, t, t\_crit)  
> Solve the set of differential equations
>
> ```
>           dx
>           -- = f(x, t)
>           dt
> ```
>
> with
>
> ```
>           x(t_0) = x_0
> ```
>
> The solution is returned in the matrix x, with each row
> corresponding to an element of the vector t. The first element
> of t should be t\_0 and should correspond to the initial
> state of the system x\_0, so that the first row of the output
> is x\_0.
>
> The first argument, fcn, is a string, inline, or function handle
> that names the function f to call to compute the vector of right
> hand sides for the set of equations. The function must have the form
>
> ```
>           xdot = f (x, t)
> ```
>
> in which xdot and x are vectors and t is a scalar.
>
> If fcn is a two-element string array or a two-element cell array
> of strings, inline functions, or function handles, the first element names
> the function f described above, and the second element names a
> function to compute the Jacobian of f. The Jacobian function
> must have the form
>
> ```
>           jac = j (x, t)
> ```
>
> in which jac is the matrix of partial derivatives
>
> ```
>                        | df_1  df_1       df_1 |
>                        | ----  ----  ...  ---- |
>                        | dx_1  dx_2       dx_N |
>                        |                       |
>                        | df_2  df_2       df_2 |
>                        | ----  ----  ...  ---- |
>                 df_i   | dx_1  dx_2       dx_N |
>           jac = ---- = |                       |
>                 dx_j   |  .    .     .    .    |
>                        |  .    .      .   .    |
>                        |  .    .       .  .    |
>                        |                       |
>                        | df_N  df_N       df_N |
>                        | ----  ----  ...  ---- |
>                        | dx_1  dx_2       dx_N |
> ```
>
> The second and third arguments specify the initial state of the system,
> x\_0, and the initial value of the independent variable t\_0.
>
> The fourth argument is optional, and may be used to specify a set of
> times that the ODE solver should not integrate past. It is useful for
> avoiding difficulties with singularities and points where there is a
> discontinuity in the derivative.
>
> After a successful computation, the value of istate will be 2
> (consistent with the Fortran version of Lsode).
>
> If the computation is not successful, istate will be something
> other than 2 and msg will contain additional information.
>
> You can use the function `lsode_options` to set optional
> parameters for `lsode`.
>
>
>
> **See also:** daspk, dassl, dasrt.

— Loadable Function: **lsode\_options** (opt, val)  
> When called with two arguments, this function
> allows you set options parameters for the function `lsode`.
> Given one argument, `lsode_options` returns the value of the
> corresponding option. If no arguments are supplied, the names of all
> the available options and their current values are displayed.
>
> Options include
>
> `"absolute tolerance"`: Absolute tolerance. May be either vector or scalar. If a vector, it must match the dimension of the state vector. `"relative tolerance"`: Relative tolerance parameter. Unlike the absolute tolerance, this parameter may only be a scalar. The local error test applied at each integration step is ``` abs (local error in x(i)) <= ... rtol * abs (y(i)) + atol(i) ``` `"integration method"`: A string specifying the method of integration to use to solve the ODE system. Valid values are "adams" "non-stiff": No Jacobian used (even if it is available). "bdf" "stiff": Use stiff backward differentiation formula (BDF) method. If a function to compute the Jacobian is not supplied, `lsode` will compute a finite difference approximation of the Jacobian matrix. `"initial step size"`: The step size to be attempted on the first step (default is determined automatically). `"maximum order"`: Restrict the maximum order of the solution method. If using the Adams method, this option must be between 1 and 12. Otherwise, it must be between 1 and 5, inclusive. `"maximum step size"`: Setting the maximum stepsize will avoid passing over very large regions (default is not specified). `"minimum step size"`: The minimum absolute step size allowed (default is 0). `"step limit"`: Maximum number of steps allowed (default is 100000).

Here is an example of solving a set of three differential equations using
`lsode`. Given the function

```
     function xdot = f (x, t)
     
       xdot = zeros (3,1);
     
       xdot(1) = 77.27 * (x(2) - x(1)*x(2) + x(1) \
                 - 8.375e-06*x(1)^2);
       xdot(2) = (x(3) - x(1)*x(2) - x(2)) / 77.27;
       xdot(3) = 0.161*(x(1) - x(3));
     
     endfunction
```

and the initial condition `x0 = [ 4; 1.1; 4 ]`, the set of
equations can be integrated using the command

```
     t = linspace (0, 500, 1000);
     
     y = lsode ("f", x0, t);
```

If you try this, you will see that the value of the result changes
dramatically between t = 0 and 5, and again around t = 305.
A more efficient set of output points might be

```
     t = [0, logspace (-1, log10(303), 150), \
             logspace (log10(304), log10(500), 150)];
```

See Alan C. Hindmarsh, ODEPACK, A Systematized Collection of ODE
Solvers, in Scientific Computing, R. S. Stepleman, editor, (1983) for
more information about the inner workings of `lsode`.
